# Genetic Modulation of HPV Infection and Cervical Lesions: Role of Oxidative Stress-Related Genes

**DOI:** 10.3390/antiox12101806

**Published:** 2023-09-27

**Authors:** Ângela Inácio, Laura Aguiar, Beatriz Rodrigues, Patrícia Pires, Joana Ferreira, Andreia Matos, Inês Mendonça, Raquel Rosa, Manuel Bicho, Rui Medeiros, Maria Clara Bicho

**Affiliations:** 1 Laboratório de Genética, Faculdade de Medicina, Universidade de Lisboa, 1649-028 Lisboa, Portugal; 2 Instituto de Saúde Ambiental (ISAMB) e Laboratório Associado TERRA, Faculdade de Medicina, Universidade de Lisboa, 1649-028 Lisboa, Portugal; 3 Instituto Bento da Rocha Cabral, 1250-047 Lisboa, Portugal; 4 Molecular Oncology & Viral Pathology Group, Research Center (CI-IPOP)/RISE@CI-IPOP, Portuguese Oncology Institute of Porto, 4200-072 Porto, Portugal; 5 Instituto de Medicina Preventiva e Saúde Pública, Faculdade de Medicina, Universidade de Lisboa, 1649-028 Lisboa, Portugal

**Keywords:** HPV infection, ICC, HSIL, oxidative stress, genetics

## Abstract

Human papillomavirus (HPV) infection is a necessary but not sufficient factor for the development of invasive cervical cancer (ICC) and high-grade intraepithelial lesion (HSIL). Oxidative stress is known to play a crucial role in HPV infection and carcinogenesis. In this study, we comprehensively investigate the modulation of HPV infection, HSIL and ICC, and ICC through an exploration of oxidative stress-related genes: *CβS*, *MTHFR*, *NOS3*, *ACE1*, *CYBA*, *HAP*, *ACP1*, *GSTT1*, *GSTM1*, and *CYP1A1*. Notably, the *ACE1* gene emerges as a prominent factor with the presence of the I allele offering protection against HPV infection. The association of *NOS3* with HPV infection is perceived with the 4a allele showing a protective effect. The presence of the *GSTT1* null mutant correlates with increased susceptibility to HPV infection, HSIL and ICC, and ICC. This study also uncovers intriguing epistatic interactions among some of the genes that further accentuate their roles in disease modulation. Indeed, the epistatic interactions between the BB genotype (*ACP1*) and DD genotype (*ECA1*) were shown to increase the risk of HPV infection, and the interaction between BB (*ACP1*) and 0.0 (*GSTT1*) was associated with HPV infection and cervical lesions. These findings underscore the pivotal role of four oxidative stress-related genes in HPV-associated cervical lesions and cancer development, enriching our clinical understanding of the genetic influences on disease manifestation. The awareness of these genetic variations holds potential clinical implications.

## 1. Introduction

Cervical carcinoma, a malignancy of the uterus, accounted for approximately 3.1% of all malignant tumors in 2020, ranking as the fourth most prevalent cancer among women and posing a significant global public health challenge [1]. While high-risk human papillomavirus (HPV) infection is a primary trigger for cervical cancer, it is crucial to recognize that HPV infection alone is insufficient to drive the development of this cancer or its precursor lesions [2,3,4]. Factors, such as early sexual activity, multiple partners, inadequate clinical follow-up, and tobacco use, contribute to its incidence [4]. The pathogenesis of HPV involves persistent infection in an environment of chronic inflammation, where oxidative stress (OS) plays a pivotal role [5,6,7,8,9,10,11]. The inflammatory response triggered by HPV recruits cells that release proinflammatory cytokines, initiating OS as part of the host defense mechanisms. This chronic inflammatory environment fosters the production of harmful reactive oxygen species (ROS) and damaging cell structures and creates conditions conducive to malignant transformation [9]. Beyond its role in carcinogenesis, OS is indispensable for completing the HPV life cycle, facilitating viral assembly [12,13]. Moreover, the incorporation of HPV into the cellular genome leads to the expression of oncoproteins, contributing to the chronic inflammation associated with cervical cancer [14,15]. Given OS’s prominent role, the genetic variations in oxidative stress-related genes may influence the host’s redox status, thereby influencing HPV infection and cancer development.

Cystathionine beta-synthase (CβS) and methylenetetrahydrofolate reductase (MTFHR) are pivotal enzymes in homocysteine metabolism. Homocysteine, an antioxidant and redox regulator, is associated with aminothiol profiles that are indicative of oxidative stress-related pathologies [16]. CβS mediates the binding of homocysteine and serine to cystathionine and harbors the common 844ins68 indel variation [17]. MTFHR converts 5,10-methylenetetrahydrofolate to 5-methyltetrahydrofolate, which is crucial for homocysteine re-methylation to methionine [18]. A common variant, C677T, results in an enzyme with reduced folate-processing capacity [19,20]. Notably, the *CβS* 844ins68 allele has been observed to normalize homocysteine and folate levels in individuals with the *MTFHR* C677T variation [21].

Nitric oxide synthase (NOS) converts L-arginine into L-citrulline to produce nitric oxide (NO), a central player in reactive nitrogen species (RNS) regulation. NO’s interactions with unpaired electrons generate ROS, such as peroxide nitrite (ONOO–), and its oxidation products may lead to cellular toxicity [9]. Crosstalk between NOS and other cellular components, including mitochondria and xanthine oxidase, can modulate ROS generation [22,23]. However, at low concentrations, intracellular NO may function as an antioxidant by promoting the cessation of reactions with lipid radicals, resulting in the formation of less reactive secondary nitrogen-containing products (LONO, LOONO) at near diffusion-limited rates. In the presence of suboptimal concentrations of L-arginine or the cofactor BH4, eNOS is functionally ‘uncoupled’ and produces superoxide anion (O2-) [24]. Even though several polymorphic forms of the *NOS3* gene have been found, one of the most important with clinical importance and associated with variations in the NO levels is the variable number of a 27 bp tandem repeat (VNTR) in intron 4 [25]. There are two major alleles, the 4b allele with 5 tandem repeats and the 4a allele with 4 repeats. This polymorphism regulates *NOS3* post-transcriptionally by altering the formation of a small interfering RNA (siRNA). In vitro studies have shown higher siRNA levels in endothelial cells containing five copies (4b), determining lower *NOS3* mRNA levels [26].

Angiotensin-converting enzyme (ACE1) plays a role in oxidative stress by mediating the production of angiotensin II, a potent oxidative stress mediator. Angiotensin II activates membrane NAD(P)H oxidases in vascular smooth muscle cells to produce ROS, such as superoxide and hydrogen peroxide, which are then involved in pleiotropic effects [27]. *ACE* I/D-variation consists of the insertion or deletion (I/D) of a 287 bp fragment in intron 16. The D allele has been associated with higher ACE activity, accounting for 47% of the total phenotypic variance of serum enzyme levels [28].

The NOX enzyme is a membrane-associated, multi-protein that produce O2- for host defense and other functions [29]. The generation of extracellular O2- through NOX is associated with oncogene activation and seems to be required for the control of cell proliferation and maintenance of the transformed state [30]. The CYBA protein is one of the membrane subunits of NOX [31]. The rs4673 polymorphism (C242T) in the *CYBA* gene causes a functional nonconservative substitution from histidine-72 to a tyrosine residue that decreases its activity [32].

Although haptoglobin is a late acute-phase protein of inflammation, it also binds to free hemoglobin released during intravascular hemolysis. Free hemoglobin can catalyze OH formation from H_2_O_2_, and heme, per se, can act as a pro-oxidant [33,34]. Two major alleles of haptoglobin exist (*Hp1* and *Hp2*), and the way their gene products interact produces three main phenotypes of haptoglobin—Hp1.1, Hp2.1, and Hp2.2—which may have an impact on the strength of the interaction with hemoglobin; Hp 2.2 is biologically less-effective in binding free hemoglobin [35].

Glutathione S-transferases (GSTs) play a vital role in detoxifying electrophilic compounds by generating free radicals. The genetic variation in the genes encoding GST enzymes is, therefore, crucial to regulating oxidative stress. The null genotype of *GSTM1/T1* results in loss of enzyme activity [36].

Acid phosphatase locus 1 (*ACP1*) is a gene that encodes a low molecular weight phosphotyrosine phosphatase (LMW-PTP), which presents two main enzymatic activities: phosphoprotein tyrosine phosphatase and flavin mononucleotide phosphatase. The *ACP1* genotype was found to directly correlate to glutathione reductase activity [37]. Two different isoenzymes have been described, the fast and slow, that arise through alternative splicing mechanisms in which either exon 3 or exon 4 is excised and the other retained, respectively. There are three common codominant alleles of *ACP1*: ACP1*A, ACP1*B, and ACP1*C. The *ACP1* alleles differ on single-nucleotide polymorphisms (SNPs), which affect both the total enzymatic activity and the ratio between isoforms F/S [38].

The cytochrome P450 proteins are monooxygenases, which catalyze many reactions involved in drug metabolism and synthesis of cholesterol, steroids, and other lipids. Cellular sources for the production of redox-active molecules may include cytochrome P450 [39]. During cellular respiration in the mitochondria, O_2_ can be reduced to its most reactive radical, O2–, through cytochrome P450 reductases [13]. *CYP1A1* encodes a member of the cytochrome P450 superfamily of enzymes. T5639C is a 3′UTR variant, probably affecting its mRNA stability. A4889G and C4887A are missense mutations already reported to be associated with a set of diseases that is oxidative related [40,41,42,43].

The association between oxidative stress-related genes, such as *CβS*, *MTFHR*, *NOS3*, *ACE1*, *CYBA*, *HAP*, *GSTT1*, *GSTM1*, *ACP1*, and *CYP1A1*, and HPV infection and cervical lesions forms the focus of our investigation.

## 2. Materials and Methods

### 2.1. Study Participants

A total of 308 women infected with HPV (172 with invasive cervical carcinoma, 31 with high-grade squamous intraepithelial lesions (HSIL), and 44 with low-grade squamous intraepithelial lesions (LSIL)) from the Portuguese Oncological Institutes in Lisbon and Oporto were included in this study. All women had cytologic and histopathologic analyses performed. HPV detection was performed using polymerase chain reaction (HPV-DNA using the PGMY09/11, [44]) and hybridization (Hybrid Capture-2, DIGENE, Gaithersburg, MD, USA) methods. The pathological group’s age ranged from 19 to 81 years with a median age of 46 years. A control group of 552 healthy Caucasian women with an age range of 19 to 80 years and a median age of 48 years was used. This population was obtained from a group of women involved in a program of exercise from the Faculty of Sport and Exercise Sciences of Lisbon. The two groups did not exhibit significant age differences (*p* = 0.111). DNA extracts were obtained between 1996 and 2003 and are now categorized as archived samples.

### 2.2. Sample Collection and Ethics

Archived samples collected between 1996 and 2003 were utilized in this study. The samples used in this study were approved for genetic studies by a scientific and committee board (Supplementary Material). Written informed consent was obtained from all participants. Due to the archived nature of the samples, re-collection of biological material from the same individuals was not feasible, resulting in variations in sample numbers (N) for different gene analyses.

### 2.3. DNA Extraction

Whole blood samples were obtained from the patients and controls. Genomic DNA was isolated from 200 μL of whole blood using the NZY Tissue gDNA isolation kit, which is a spin column silica-based method. The samples were processed following the manufacturer’s instructions.

### 2.4. Genotyping

Genotyping was performed with PCR-based methods. The primers and PCR conditions are listed below.
**Gene****PCR Primers****PCR Conditions***CβS*5′GCAGTTGTTAACGGCGGTATTG3′ 5′GCCGGGCTCTGGACTCGACCTA3′40 cycles 94 °C–30 s 60 °C–30 s 72 °C–40 s*MTHFR*5′TGAAGGAGAAGGTGTCTGCGGGA3′ 5′AGGACGGTGCGGTGAGAGTG3′35 cycles 94 °C–30 s 61 °C–30 s 72 °C–45 s*NOS3*5′AGGCCCTATGGTAGTGCCTTT3′ 5′TCTCTTAGTGCTGTGGTCAC3′30 cycles 94 °C–30 s 55 °C–30 s 72 °C–45 s*ACE1*5′GCCCTGCAGGTGTCTGCAGCATGT3′ 5′ GGATGGCTCTCCCCGCCTTCTCTC3′30 cycles 94 °C–60 s 60 °C–30 s 72 °C–30 s*CYBA*5′TGCTTGTGGGTAAACCAAGGCCGGTG3′ 5′AACACTGAGGTAAGTGGGGGTGGCTCCGT3′35 cycles 94 °C–43 s 54 °C–60 s 72 °C–30 s*ACP1*5′CGATCACCCATTGCAGAAG3′ 5′CCATGATTTCTTAGGCAGCTC3′35 cycles 94 °C–30 s 54 °C–45 s 72 °C–45 s*GSTT1* and *GSTM1*5′GCCATCTTGTGCTACATTGCCCG3′, 5′ATCTTCTCCTCTTCTGTCTCCCC3′, 5′TTCTGGATTGTAGCAGATCATGCCC3′, 5′TTCCTTACTGGTCCTCACATCTC3′ 5′TCACCGGATCATGGCCAGCA3′40 cycles 94 °C–45 s 58 °C–45 s 72 °C–45 s*CYP1A1* rs49868845′CAGTGAAGAGGTGTAGCCGC3′ 5′TAGGAGTCTTGTCTCATGCC 3′30 cycles 94 °C–60 s 61 °C–60 s 72 °C–60 s*CYP1A1* rs1048943 rs17998145′CTGTCTCCCTCTGGTTACAGGAAGC3′ 5′TTCCACCCGTTGCAGCAGGATAGCC3′35 cycles 94 °C–45 s 64 °C–45 s 72 °C–75 s

*CβS Genotyping—−/+*

An amplification with PCR, flanking the 844ins68bp variation, was performed. The resulting amplicons were visualized in an agarose gel with 252 bp representing the allele lacking an insertion and 320 bp representing the allele with an insertion.

*MTHFR Genotyping—rs1801133*

An amplification with PCR, flanking the C677T variation, was performed. An amplicon of 198 bp was visualized in an agarose gel. Subsequent restriction with *Hinf* I facilitated genotyping: CC-198 bp; CT-198, 175, and 23 bp; TT-175 and 23 bp.

*NOS3 Genotyping—4b/4a*

An amplification with PCR, flanking the 4a/4b (27bp-VNTR) variation, was performed. The amplicons were visualized in an agarose gel with 420 bp representing the allele with an additional VNTR (b) repeat and 393 bp representing the allele without an additional VNTR repeat (a).

*ACE1 Genotyping—rs4646994*

An amplification with PCR, flanking the I/D (insertion–deletion) variation, was performed. The amplicons were visualized in an agarose gel with 319 bp representing the allele with the deletion (D) and 597 bp representing the allele with the insertion (I).

*CYBA Genotyping—rs4673*

An amplification with PCR, flanking the C-242T variation, was performed. An amplicon of 348 bp was visualized in an agarose gel. Subsequent restriction with *Rsa*I allowed for the facilitated genotyping: CC-348 bp; CT-348, 188, and 160 bp; TT-188 and 160 bp.

*HAP Genotyping—1/2*

Hap genotyping was determined using the Hp phenotype (Hp–Hb complexes) with PAGE electrophoresis and peroxidase staining that followed a modified version of Linke’s method (1984) [45].

*ACP1 Genotyping—A/B/C*

An amplification with PCR, flanking the A/B/C variation, was performed. An amplicon of 400 bp was visualized in an agarose gel. Subsequent restriction with *Bsh*136I and *Msp*A1I facilitated genotyping: *Bsh1*36I generated two fragments of 225 bp and 175 bp when alleles A or B, while *Msp*A1I generated two fragments of 328 bp and 72 bp for alleles B and C.

*GSTT1 and GSTM1 Genotyping—1/0*

An amplification with multiplex PCR, flanking each gene, was performed. The amplicons were visualized in an agarose gel, where 480 bp represented the presence of the *GSTT1* gene, 230 bp represented the presence of the *GSTM1* gene, and the 157 bp band served as the positive control for the amplification. A null allele was identified by the absence of the corresponding gene amplicon and the presence of the control amplicon.

*CYP1A1 Genotyping—rs4986884|rs1048943|rs1799814*

An amplification with PCR, flanking the T5639C (rs4986884) variation, was performed. An amplicon of 340 bp was visualized in an agarose gel. Subsequent restriction with *Msp*I facilitated genotyping: two fragments of 200 bp and 140 bp identified allele C, while the absence of restriction identified allele T. An amplification with PCR, flanking the A4889G (rs1048943) variation and C4887A (rs1799814), was performed. An amplicon of 204 bp was visualized in an agarose gel. Restriction with *Bsr*DI identified two fragments of 149 and 55 bp for allele A (rs1048943), while the absence of restriction identified allele G. Restriction with *Bsa*I identified two fragments of 139 and 65 bp for allele C (rs1799814), while the absence of restriction identified allele A.

### 2.5. Statistical Analysis

All statistical tests were conducted using SPSS 28.0 software. Group differences were assessed using Pearson’s chi-square or Fisher tests. When more than 20% of cells had an expected count inferior to 5, correction for chi-square was conducted using Fisher’s Exact test (2 × 2 tables) or the Monte Carlo simulation method for chi-square (other tables). Statistical significance was defined as a *p*-value < 0.005 after correction for multiple testing using the Bonferroni adjustment.

## 3. Results

We commenced our analysis by investigating the association between HPV infection and ten distinct genes (*CβS*, *MTHFR*, *NOS3*, *ACE1*, *CYBA*, *HAP*, *GSTT1*, *GSTM1*, *ACP1*, and *CYP1A1*).

### 3.1. Analysis Using the Codominant Model

Under the codominant model, we initially compared the distribution of genotypes across HPV-infected individuals and controls (Table 1).

Among the genes studied, only *ACE1* displayed dissimilar genotype distributions between the two female populations (*p* = 0.003). Notably, due to the inability of our *GSTM1*/*GSTT1* genotyping methodology to distinguish between 1/1 and 1/0, this gene was excluded from the codominant analysis.

### 3.2. Analysis Using the Allelic Model

For a clearer understanding of the risk or protective effects of each allele, we examined the allele distribution between the two populations whenever feasible (Table 2). Notably, *NOS3* (*p* = 0.003) and *ACE1* (*p* = 0.002) exhibited distinct allele distributions. In the case of *NOS3*, allele 4a demonstrated a protective factor (OR = 0.470, CI 0.283–0.781). Regarding *ACE1*, allele I displayed a protective effect (OR = 0.572, CI 0.403–0.813).

### 3.3. Analysis Using Dominant, Overdominant, and Recessive Models, or Functional Interest

In cases where feasible, we evaluated the genotype distribution using the dominant, overdominant, and recessive models (Table 3). Notably, for *ACP1*, which comprises more than two possible alleles, two distinct analyses were conducted: one contrasted a single genotype against all others, and the other grouped the genotypes based on functional characteristics. The genotypes were aggregated according to fast/slow ratio ((AA and AB and BB) < (AC and BC)), fast isoform concentration ((AA and AC) < (AB and BC) < (BB)), slow isoform concentration ((AA and AB) < (BB and AC) < (BC)), or total activity ((BB and AB)/(AA and BC and AC)). These isoforms possess differing catalytic and molecular properties, potentially serving distinct cellular functions [45,46,47].

The genotype distributions varied for the *ACE1* and *GSTT1* genes. In the case of *ACE1*, the presence of allele I (II and ID) were associated with protection (*p* < 0.001, OR = 0.469, CI 0.302–0.728). Conversely, the *GSTT1* null allele (0/0) was considered a risk factor (*p* < 0.001; OR = 2.691, CI 1.510–4.794).

### 3.4. Analysis Involving the Most Severe Cytological Phenotypes

Finally, we performed a focused analysis involving samples characterized by cytological phenotypes, narrowing our scope to invasive cervical cancer (ICC) or the most severe phenotypes, namely high-grade squamous intraepithelial lesion (HSIL) plus ICC. Analyzing the genotype distribution within the lesions further underscored the influence of *GSTT1* in this disease context. Once again, the genotype of *GSTT1* exhibited significant associations with ICC (*p* = 0.003, OR = 2.759, CI 1.394–5.459) and HSIL and ICC (*p* = 0.001, OR = 2.814, CI 1.475–5.366) (Table 4).

We also tested for differences in the genotype distributions between LSIL, HSIL, and ICC, but found no significant results (Table 5).

### 3.5. Epistatic Analysis

Subsequently, we delved into the most promising epistatic interactions, including those between homozygous genotypes or genotypes featuring the presence of one of the alleles (Table 6). Notably, concerning HPV infection, an increased risk was observed for the *ACP1*(BB)-*ACE1*(DD)-epistatic interaction when compared to each individual genotype (OR = 2.643, CI 1.335–5.232 for *ACE1*(DD)-*ACP1*(BB) compared to OR = 2.131, CI 1.373–3.309 for *ACE1*(DD) and no risk for *ACP1*(BB)). A similar trend was noted for the epistatic interaction *ACP1*(BB)-*GSTT1*(0.0) in HPV infection (OR = 5.707, CI 1.665–19.565 for *ACP1*(BB)-*GSTT1*(0.0) compared to no risk for *ACP1*(BB) and OR = 2.691, CI 1.510–4.794 for *GSTT1*(0.0)). This last epistasis was also present when the analysis was applied to the population with the most severe lesions (HSIL and IC), in this case an OR = 9.455 (CI 2.615–34.187) was much higher than the risk of having only *GSTT1*(0.0) (OR = 2.759, CI 1.394–5.459), while the BB genotype from *ACP1* presented no risk alone.

## 4. Discussion

In this study, our investigation revealed associations between HPV infection or cervical lesions and four of the ten candidate genes under consideration.

The gene most consistently associated with HPV infection was *ACE1*, where the I allele emerged as a protective factor. The allele’s protective effect could be attributed to its connection with a lower ACE activity and subsequent reduction in ROS levels given that angiotensin II activates NAD(P)H oxidases [27,28]. Previously, an enhanced ACE activity was verified in tumor progression in cervical carcinoma [48,49]. Regarding other types of cancer, the following has been described in gastric and colorectal cancer: a higher *ACE1* expression in the tumor microenvironment compared to healthy tissues [50], a higher susceptibility to prostate cancer in carriers of the D allele [51], and a lower incidence of breast cancer for the genotypes with lower gene expression and for women receiving treatment with iACEs [52]. Although we could not find an association of this gene with cervical lesions, we know that HPV infection is necessary for carcinoma development. One possibility is that the limitations on our sample size concerning lesions (with available genotype) may be hindering a positive result. Indeed, our analysis shows a trend to associate the presence of the I allele with protection of cervical lesions (Supplementary Table S1).

Our study also revealed an association between *NOS3* and HPV infection. Other studies have indicated that lower siRNA levels in endothelial cells carrying five copies (4a) result in elevated *NOS3* mRNA levels [53]. As a vital messenger, nitric oxide (NO) plays pivotal roles in various pathophysiological processes, including neurotransmission, vascular homeostasis, inflammation, and immune responses [54]. Notably, NO is integral to localized immune defense and HPV eradication [55]. In addition, it is known that HPV infection increases NO release [56,57,58]. NO is produced by epithelial, stromal, and endothelial cells of the uterine cervix [56,59,60,61]; therefore, genetic variation in *NOS3* that favors NO production may be an advantage for HPV clearance. Our findings align with this concept as the 4a variation emerged as a protective factor against HPV infection, suggesting that genetic variations favoring NO production may aid HPV clearance. However, the role of NO in carcinogenesis is complex, as its consequences are dose-dependent and time-sensitive. While low NO levels promote cell proliferation and anti-apoptotic responses, high levels induce cycle arrest, apoptosis, and senescence through oxidative and nitrosative stresses [22,62]. Although we were not able to associate *NOS3* with cancer, phytochemical-induced apoptosis has been linked to the NO signaling pathway activation in cervical cancer [63].

Concerning the *GST* genes, the corresponding proteins play a crucial role in the mechanisms against oxidative stress. The homeostasis of a powerful antioxidant, glutathione, is essentially regulated by GST activity. In addition, GST proteins are responsible for the high-capacity metabolic inactivation of electrophilic compounds and toxic substrates. *GST*-null genotypes result in the loss of the enzyme’s ability to bind genotoxic substrates, leading to a decreased detoxification and an increased tumorigenesis [64,65]. Our results show the association of the *GSTT1*-null genotype with HPV, lesions, and cancer. Also, de Carvalho et al. [66] verified a significant increase in the *GSTT1*-null genotype in uterine cervix adenocarcinoma, while the *GSTM1*-null variant was not implicated in this disease. However, meta-analysis studies revealed that the *GSTM1*-null variant caused cervix lesions, especially among HPV infection in Indian and Chinese populations [67,68]. Also, it was reported that women who are HPV-positive and who carry both null genotypes present an increased risk of cervical carcinoma developing before the age of 40 years [69]. Our results do not show an association of *GSTM1* or double mutants with cervical cancer or precursor lesions; *GST*-null mutants have been reported to be associated with oncologic diseases in general, though [70].

Both *CβS* and *MTHFR* did not exhibited associations with HPV infection or major lesions. Meta-analyses, other reviews, and original papers have been describing associations of this *MTHFR* variant with several types of cancers, such as ovarian cancer, breast cancer, colorectal cancer, bladder cancer, lung cancer, childhood acute lymphoblastic leukemia [71,72,73,74,75,76], and even cervical cancer [77,78,79,80,81]. However, such associations remain contentious [82,83,84,85], potentially due to population differences [81]. Concerning *CβS*, our bibliographic survey did not reveal any study of this gene in HPV infection, cervical cancer, or precursor lesions. Nevertheless, this variant has been associated with breast cancer and exhibits altered expression in various cancer forms [86,87].

Our investigation highlighted the *ACP1* gene’s relevance. However, the results presented in this work are puzzling since no relevant result concerning functionality was achieved, and the BB genotype was found as a risk factor for HPV infection and severe lesions if in epistasis. Notably, the BB genotype in epistasis with DD (from *ACE1*) or 0.0 (from *GSTT1*) indicated risks for these phenotypes. This underscores the interplay of genetic backgrounds in disease contribution. Although lacking prior associations with HPV infection or cervical lesions, the *ACP1* genetic variation has been linked to other cancer types [88,89]. Curiously, a positive correlation between the amount of the enzyme in human tumors and a poor prognosis was verified [90].

The *CYBA* gene revealed no association with HPV infection, cervical lesions, or cancer. However, CYBA’s involvement has been previously verified in cervical cancer [91], and the T allele has been associated with reduced O2- generation in the phagocytic respiratory burst [92]. This variant was already described in association with other types of cancer [93,94].

In our study, *HAP* was not implicated in tumorigenesis or HPV infection. However, it is known that Haptoglobin’s role in binding free hemoglobin contributes to oxidative damage prevention [95]. Regarding cervical cancer, there are controversial results. While the incidence of the genotype 1.1 was first shown to be lower in Chinese women [96], Mahmud et al. [97] reported that women carrying the *Hp* 1 allele, particularly in homozygosity, had an increased risk of developing invasive neoplastic lesions. A study carried out at the University of Ghana also obtained similar results, concluding that individuals with the Hp 2.2 phenotype had a lower risk of developing cervical cancer compared to Hp 1.1 individuals [98]. There is evidence that Hp 2.2 may play a more important role in sequestering iron at the tissue level, which appears to occur exclusively in Hp2.2 subjects [99]. Nowadays, it is believed that Hp plasma concentration and phenotype can modulate the individual predisposition of a person to various diseases, including several types of cancer; because of that, Hp is frequently the subject of research as a potential biomarker [97].

Our investigation did not reveal significant associations for the *CYP1A1* gene. There is at least one study able to associate *CYP1A1* with susceptibility to HPV infection [100], and all of our variants (T5639C, A4889G, and C4887A) were found to be involved in cervical cancer in other studies [101,102,103,104,105,106,107,108]. Similar to our case, the A4889G variant was not associated with cervical cancer in an Indian population [108]; still, our results seem to not fit the trend of most studies.

The discrepancies between our study and others could reflect unique genomic features of the Portuguese population. Being one of the oldest countries in Europe, Portugal identity results from the combination of the histories of Iberian tribes, Celtic peoples, the Roman Empire, Germanic kingdoms, Muslim invasions, Christian reconquer, exploration of the “New World”, and emigration and immigration fluxes. Indeed, the Portuguese population has a history of miscegenation with other populations, resulting in a unique genomic background.

As limitations to this work, we consider the lack of information on HPV genotyping, sexually transmitted diseases (HIV, chlamydia, Herpes), diet on folic acid, tabaco (immune suppressor), sex hormone intake, and medication with ACE inhibitors. These factors could potentially act as confounders, though gathering such information from archival samples is challenging.

## 5. Conclusions

In conclusion, our study underscores the importance of four oxidative stress-related genes in HPV infection, cervical lesions, and invasive cervical cancer. While governmental vaccine programs are mitigating this issue in some regions, underdeveloped countries still grapple with HPV-related public health challenges. Further research, especially focusing on functional aspects, will be essential to unravel the molecular mechanisms involved. The clinical significance of these genetic variations should not be overlooked, as they contribute to disease susceptibility in this context.

## Figures and Tables

**Table 1:** Comparison of genotype distribution using the codominant model—HPV vs. Control.

Genes		HPV	Controls	*p*-Value
		N (%)	N (%)	
*CβS*	−/−	46 (93.9%)	220 (83.0%)	0.077 ^a^
	+/−	2 (4.1%)	42 (15.8%)	
	+/+	1 (2.0%)	3 (1.1%)	
*MTHFR*	CC	53 (44.5%)	200 (48.5%)	0.546 ^b^
	CT	54 (45.4%)	164 (39.8%)	
	TT	12 (10.1%)	48 (11.7%)	
*NOS3*	4b4b	80 (79.2%)	125 (66.1%)	0.018 ^b^
	4a4b	20 (19.8%)	50 (26.5%)	
	4a4a	1 (1.0%)	14 (7.4%)	
*ACE1*	DD	70 (64.2%)	176 (45.7%)	0.003 ^b,^*
	ID	29 (26.6%)	159 (41.3%)	
	II	10 (9.2%)	50 (13.0%)	
*CYBA*	CC	46 (51.7%)	34 (38.2%)	0.190 ^b^
	CT	36 (40.4%)	47 (52.8%)	
	TT	7 (7.9%)	8 (9.0%)	
*HAP*	2.2	72 (36.5%)	129 (38.7%)	0.163 ^b^
	2.1	78 (39.6%)	147 (44.1%)	
	1.1	47 (23.9%)	57 (17.1%)	
*ACP1*	AA	3 (4.9%)	27 (11.0%)	0.299 ^b^
	BB	30 (49.2%)	87 (35.4%)	
	AB	21 (34.4%)	97 (39.4%)	
	AC	3 (4.9%)	16 (6.5%)	
	BC	4 (6.6%)	19 (7.7%)	
*CYP1A1* T5639C	TT	41 (70.7%)	98 (81.0%)	0.121 ^b^
	TC	17 (29.3%)	23 (19.0%)	
*CYP1A1* A4889G	AA	43 (87.8%)	117 (92.1%)	0.387 ^c^
	AG	6 (12.2%)	10 (7.9%)	
*CYP1A1* C4887A	CC	39 (90.7%)	109 (86.5%)	0.472 ^b^
	CA	4 (9.3%)	17 (13.5%)	

^a^ Monte Carlo simulation method for chi-square; ^b^ Chi-square test; ^c^ Fisher’s exact test; * Significant.

**Table 2:** Comparison of allele distribution using the allelic model—HPV vs. Control.

Genes			HPV	Controls	*p*-Value	OR (CI)
			N (%)	N (%)		
*CβS*		Allele −	94 (95.9%)	482 (90.9%)	0.101 ^a^	
		Allele +	4 (4.1%)	48 (9.1%)		
*MTHFR*		Allele C	160 (67.2%)	564 (68.4%)	0.722 ^a^	
		Allele T	78 (32.8%)	260 (31.6%)		
*NOS3*		Allele 4b	180 (89.1%)	300 (79.4%)	0.003 ^a,^*	0.470 (0.283–0.781)
		Allele 4a	22 (10.9%)	78 (20.6%)		
*ACE1*		Allele D	169 (77.5%)	511 (66.4%)	0.002 ^a,^*	0.572 (0.403–0.813)
		Allele I	49 (22.5%)	259 (33.6%)		
*CYBA*		Allele C	128 (71.9%)	115 (64.6%)	0.139 ^a^	
		Allele T	50 (28.1%)	63 (35.4%)		
*HAP*		Allele 2	222 (56.3%)	405 (60.8%)	0.153 ^a^	
		Allele 1	172 (43.7%)	261 (39.2%)		
*ACP1*		Allele A	30 (24.6%)	167 (33.9%)	0.091 ^a^	
		Allele B	85 (69.7%)	290 (58.9%)		
		Allele C	7 (5.7%)	35 (7.1%)		
*CYP1A1*	T5639C	Allele T	99 (85.3%)	219 (90.5%)	0.148 ^a^	
		Allele C	17 (14.7%)	23 (9.5%)		
	A4889G	Allele A	92 (93.9%)	244 (96.1%)	0.397 ^b^	
		Allele G	6 (6.1%)	10 (3.9%)		
	C4887A	Allele C	82 (95.3%)	235 (93.3%)	0.487 ^a^	
		Allele A	4 (4.7%)	17 (6.7%)		

^a^ Chi-square test, ^b^ Fisher´s exact test, * Significant.

**Table 3:** Comparison of genotype distribution using the dominant, overdominant, and recessive models—HPV vs. Controls.

Genes ^1^		HPV			Controls			*p*-Value	OR (CI)
		N (%)			N (%)				
*CβS*	+/+ and +/− vs. −/−	3 (6.1%)	46 (93.9%)		45 (17.0%)	220 (83.0%)		0.052 ^a^	
	+/− vs. −/− and +/+	2 (4.1%)	47 (95.9%)		42 (15.8%)	223 (84.2%)		0.029 ^a^	
	+/+ vs. −/+ and +/+	1 (2.0%)	48 (98.0%)		3 (1.1%)	262 (98.9%)		0.495 ^b^	
*MTHFR*	TT and TC vs. CC	66 (55.5%)	53 (44.5%)		212 (51.5%)	200 (48.5%)		0.441 ^a^	
	CT vs. CC and TT	54 (45.4%)	65 (54.6%)		164 (39.8%)	248 (60.2%)		0.276 ^a^	
	TT vs. CC and CT	12 (10.1%)	107 (89.9%)		48 (11.7%)	364 (88.3%)		0.634 ^a^	
*NOS3*	4a4a and 4a4b vs. 4b4b	21 (20.8%)	80 (79.2%)		64 (33.9%)	125 (66.1%)		0.020 ^a^	
	4a4b vs. 4a4a and 4b4b	20 (19.8%)	81 (80.2%)		50 (26.5%)	139 (73.5%)		0.207^a^	
	4a4a vs. 4b4b and 4a4b	1 (1.0%)	100 (99.0%)		14 (7.4%)	175 (92.6%)		0.019 ^a^	
*ACE1*	II and ID vs. DD	39 (35.8%)	70 (64.2%)		209 (54.3%)	176 (45.7%)		<0.001 ^a,^*	0.469 (0.302–0.728)
	ID vs. DD and II	29 (26.6%)	80 (73.4%)		159 (41.3%)	226 (58.7%)		0.005 ^a^	
	II vs. DD and ID	10 (9.2%)	99 (90.8%)		50 (13.0%)	335 (87.0%)		0.282 ^a^	
*CYBA*	TT and TC vs. CC	43 (48.3%)	46 (51.7%)		55 (61.8%)	34 (38.2%)		0.071 ^a^	
	CT vs. CC and TT	36 (40.4%)	53 (59.6%)		47 (52.8%)	42 (47.2%)		0.098 ^a^	
	TT vs. TC and CC	7 (7.9%)	82 (92.1%)		8 (9.0%)	81 (91.0%)		0.787 ^a^	
*HAP*	2.1 and 1.1 vs. 2.2	125 (63.5%)	72 (36.5%)		204 (61.3%)	129 (38.7%)		0.615 ^a^	
	2.1 vs. 2.2 and 1.1	78 (39.6%)	119 (60.4%)		147 (44.1%)	186 (55.9%)		0.306 ^a^	
	1.1 vs. 2.2 and 2.1	47 (23.9%)	150 (76.1%)		57 (17.1%)	276 (82.9%)		0.059 ^a^	
*ACP1*	AA vs. BB and CC and AB and AC and BC	3 (4.9%)	58 (95.1%)		27 (11.0%)	219 (89.0%)		0.154 ^a^	
	BB vs. AA and CC and AB and AC and BC	30 (49.2%)	31 (50.8%)		87 (35.4%)	159 (64.6%)		0.047 ^a^	
	AB vs. AA and BB and CC and AC and BC	21 (34.4%)	40 (65.6%)		97 (39.4%)	149 (60.6%)		0.472 ^a^	
	AC vs. AA and BB and CC and AB and BC	3 (4.9%)	58 (95.1%)		16 (6.5%)	230 (93.5%)		0.775 ^b^	
	BC vs. AA and BB and CC and AB and AC	4 (6.6%)	57 (93.4%)		19 (7.7%)	227 (92.3%)		1 ^b^	
	AA and AB and BB vs. AC and BC	54 (88.5%)	7 (11.5%)		211 (85.8%)	35 (14.2%)		0.576 ^a^	
	AA and AC and BC vs. BB and AB	10 (16.4%)	51 (83.6%)		62 (25.2%)	184 (74.8%)		0.146 ^a^	
	AA and AC vs. AB and BC vs. BB	6 (9.8%)	25 (41.0%)	30 (49.2%)	43 (17.5%)	116 (47.2%)	87 (35.4%)	0.098 ^a^	
	AA and AB vs. BB and AC vs. BC	24 (39.3%)	33 (54.1%)	4 (6.6%)	124 (50.4%)	103 (41.9%)	19 (7.7%)	0.225 ^a^	
*GSTT1*	0/0 vs. 1/1 and 1/0	35 (36.5%)	61 (63.5%)		29 (17.6%)	136 (82.4%)		<0.001 ^a,^*	2.691 (1.510–4.794)
*GSTM1*	0/0 vs. 1/1 and 1/0	58 (53.2%)	51 (46.8%)		73 (44.0%)	93 (56.0%)		0.134 ^a^	

^a^ Chi-square test; ^b^ Fisher’s exact test; ^1^ first row—dominant model; second row—over dominant model; third row—recessive model (except *ACP1*); * Significant.

**Table 4:** Comparison of genotype distribution—ICC vs. Control and HSIL and ICC vs. Control.

		ICC	Controls	*p*-Value *	OR (CI)
		N (%)	N (%)		
*GSTT1*		20	29	0.003	2.759 (1.394–5.459)
	00	(37.0%)	(17.6%)		
		34	136		
	01&11	(63.0%)	(82.4%)		
		**HSIL and ICC**	**Controls**	*p*-Value *	**OR** **(CI)**
		**N** **(%)**	**N** **(%)**		
*GSTT1*		24	29	0.003	2.759 (1.394–5.459)
	00	(37.5%)	(17.6%)		
		40	136		
	01&11	(62.5%)	(82.4%)		

* Chi-square test.

**Table 5:** Comparison of genotype distribution—LSIL/HSIL/ICC.

Gene		LSIL N (%)	HSIL N (%)	ICC N (%)	*p*-Value
	−/−	7 (87.5)	2 (100.0)	20 (95.2)	
*CβS*	+/−	0 (0.0)	0 (0.0)	1 (4.8)	0.548 ^a^
	+/+	1 (12.5)	0 (0.0)	0 (0.0)	
	CC	16 (55.2)	3 (27.3)	28 (41.2)	
*MTHFR*	CT	8 (27.6)	7 (63.6)	34 (50.0)	0.182 ^a^
	TT	5 (17.2)	1 (9.1)	6 (8.8)	
	4a4a	0 (0.0)	1 (12.5)	1 (0.0)	
*NOS3*	4a4b	4 (28.6)	1 (12.5)	12 (24.0)	0.072 ^b^
	4b4b	10 (71.4)	6 (75.0)	38 (76.0)	
	DD	20 (62.5)	11 (68.8)	31 (63.9)	
*ACE1*	ID	11 (34.4)	4 (25.0)	11 (22.4)	0.424 ^a^
	II	1 (3.1)	1 (6.3)	7 (14.3)	
	CC	7 (38.9)	10 (71.4)	18 (50.0)	
*CYBA*	CT	11 (61.1)	3 (21.4)	15 (41.7)	0.190 ^a^
	TT	0 (0.0)	1 (7.1)	3 (8.3)	
	1.1	6 (20.7)	6 (25.0)	32 (25.0)	
*HAP*	2.1	11 (37.9)	4 (16.7)	55 (43.0)	0.101 ^b^
	2.2	12 (41.4)	14 (58.3)	41 (32.0)	
	AA	0 (0.0)	0 (0.0)	1 (3.6)	
	AB	10 (52.6)	3 (33.3)	7 (25.0)	
*ACP1*	AC	0 (0.0)	0 (0.0)	3 (10.7)	0.406 ^a^
	BB	9 (47.4)	5 (55.6)	15 (53.6)	
	BC	0 (0.0)	1 (11.1)	2 (7.1)	
*CYP1A1* T5639C	TT	11 (64.7)	7 (87.5)	21 (70.0)	0.551 ^a^
	TC	6 (35.3)	1 (12.5)	9 (29.1)	
*CYP1A1* A4889G	AA	14 (87.5)	7 (87.5)	19 (86.4)	1.000 ^a^
	AG	2 (12.5)	1 (12.5)	3 (13.6)	
*CYP1A1* C4887A	CC	12 (75.0)	7 (100.0)	17 (100.0)	0.034 ^a^
	CA	4 (25.0)	0 (0.0)	0 (0.0)	
*GSTT1*	0/0	6 (27.3)	4 (40.0)	20 (37.0)	0.034 ^a^
	1/1&1/0	16 (72.7)	6 (60.0)	34 (63.0)	
*GSTM1*	0/0	15 (55.6)	10 (83.3)	28 (46.7)	0.675 ^b^
	1/1&1/0	12 (44.4)	2 (16.7)	32 (53.3)	

^a^ Monte Carlo simulation method for chi-square; ^b^ Chi-square test.

**Table 6:** Comparison of genotype distribution in epistatic interactions.

Genes		HPV	Controls	*p*-Value ^a^	OR (CI)
		N (%)	N (%)		
*ACP1-ACEI*	BB-DD Others	19	28	0.004 ^a^	2.643 (1.335–5.232)
		(33.3%)	(15.9%)		
		38	148		
		(66.7%)	(84.1%)		
*ACP1-GSTT1*	BB-0.0	9	4	0.004 ^b^	5.707 (1.665–19.565)
		(18.0%)	(3.7%)		
	Others	41	104		
		(82.0%)	(96.3%)		
		**HSIL and ICC**	**Controls**		
*ACP1-GSTT1*	BB-0.0	8	4	<0.001 ^b^	9.455 (2.615–34.187)
		(26.7%)	(3.7%)		
	Others	22	104		
		(73.3%)	(96.3%)		

^a^ Chi-square test, ^b^ Fisher’s exact test.

## Data Availability

The data presented in this study are available on request from the corresponding author.

## Supplementary Materials

The following supporting information can be downloaded at: https://www.mdpi.com/article/10.3390/antiox12101806/s1, File S1: Pareceres da comissão de ética e científica HPV; File S2: lei de 2005—comissão de ética; Table S1: Comparison of genotypes distribution—HSIL&ICC vs Control.
