# Supplementary material for: Genetic Modulation of HPV Infection and Cervical Lesions: Role of Oxidative Stress-Related Genes
Source: Antioxidants (Basel). 2023 Sep 27;12(10):1806. doi: 10.3390/antiox12101806 (PMC10604255; doi:10.3390/antiox12101806)
Supplement: Supplementary file 1 [file antioxidants-12-01806-s001.zip › antioxidants-2525416-File S1 pareceres da comissão de ética e científica HPV.pdf]

INSTITUTO PORTUGUÊS DE ONCOLOGIA
FRANCISCO GENTIL
PALHAVÃ
LISBOA

Lisboa, I.P.O.F.G., 1992, Julho 02

Exmª. Senhora
Drª. Isabel Cabral
Responsável pela Unidade de
Ginecologia

Dir/126/92

Tenho o prazer de a informar que o ensaio "Polimorfismo de acetilação hepática de neoplasias do endométrio e do ovário para determinação da predisposição genética", pode iniciar-se.

Junto cópia dos pareceres das Comissões Científica e de Ética que justificam esta decisão.

O DIRECTOR DO CENTRO

DOUTOR EDWARD LIMBERT

Em anexo: Modelo de Consentimento Informado a utilizar.

INSTITUTO PORTUGUES DE ONCOLOGIA
FRANCISCO GENTIL
PALHAVÃ
LISBOA

Lisboa, 14 de Abril de 1992.

Ex.mo Senhor Director do
Instituto Português de Oncologia,
Centro de Lisboa.

Os membros da Comissão Científica não vêem inconveniente para o Instituto na aderência ao estudo dos "Polimorfismos de Acetilação hepática de neoplasias malignas do endométrio e do ovário para determinação da predisposição genética", proposto pela Dra. Isabel Cabral.

O Presidente da Comissão Científica,

(Professor J. Carmo Pereira)

P A R E C E R

Refª - Polimorfismo de Acetilação hepática de neoplasias malignas do endométrio e do ovário para determinação da predisposição genética.

Protocolo apresentado pela Drª Isabel Cabral

Parecer do Sr.Prof.José Conde:

"Considerando o parecer favorável da Comissão Científica, relativamente a este Protocolo, na forma sucinta como está apresentado pela Drª Isabel Cabral, a Comissão de Ética nada tem a opôr.
Recomenda-se que cada doente autorize, nos termos do Consentimento Informado - Tratamento.
Junto alguns exemplares deste Modelo, já que ainda não se encontra distribuído o Modelo aprovado pela Direcção".

Nome -- Data -----------------

Data Nascimento ----------- Raça ----- Altura ----- Peso ------ Processo --------------

CARACTERISTICAS FAMILIARES

T. Vulva ------ T. Colo ----- T. Endométro ------ T. Ovário ------ Outros Tumores -----------

CARACTERISTICAS CLÍNICAS

HTA ----- Diabetes Melletos ----- Obsidade ----- Hirsotismo ----- Menarca ----- Ciclos -------

I. A. S. ----- Nº parceiros ----- Ag. infecciosos ----- I.O.---------------- Menorrag.-------

Metrorragias ------ Coitorragias ----- Terap. hormonal ------ Duração ------ Menopausa---------

Metrorragias pos-menopausa ---

DIAGNÓSTICO

Colpocitologia ------ Colposcopia ---

HPV -----Tipagem ------- Outros Ag. infecciosos--

Histeroscopia -------- Histerometria ------- Biópsia ---------------------------------------

D.H. Vagina------------------------------------- Grau Diferenciação -------------------------

D.H. Colo ------------------------------------- Grau Difernеciaçaõ --------------------------

D.H. Endocolo --------------------------------- Grau Diferenciação --------------------------

D.H. Endométrio ------------------------------- Grau Diferenciação --------------------------

Citometria de Fluxo --

Acetilação ---

TERAPEUTICA

Conização ------ Laser ------ Curieterapia ------- Rad. Externa--------Quimioterapia-----------

Hist. Total + AB ------ Hist. + AB ----- Hist. R. + Celulect. P. ------ Hist.T. + AB + A.

+ Omentect. ------ Citologia perit. -------Biópsias ------ Vulvectomia simples --------------

Vulvectomia radical + Celulectomia -----------------------

RESULTADO

Classificação do Estadiamento do Tumor (FIGO)

T1-----------

No-----------

Mo-----------

COMISSÃO DE ÉTICA

ESQUEMA DUMA PROTOCOLO TERAPÊUTICO

No essencial, são estes os parâmetros a integrar:

1. Fundamentos científicos do Protocolo terapêutico
2. Finalidades
3. Selecção dos doentes (como e quantos)
4. Diagrama do Protocolo
5. Programas Terapêuticos
6. Acções relativas a eventual toxicidade
7. Dados clínicos e laboratoriais necessários
8. Critérios para avaliar a acção terapêutica
9. Considerações estatísticas
10. Consentimento informado
11. Modêlo para registo de dados
12. Bibliografia
13. Quem orienta, colaboradores e meios de comunicação

À
Comissão de Investigação do Instituto Português de Oncologia Dr. Francisco Gentil (Lisboa)

Lisboa 27/X/97

Venho por este meio informar que se encontra ainda em elaboração um projecto de estudo no serviço de Ginecologia deste Instituto, em colaboração com um Serviço do exterior - Laboratório de Genética da FML com o título **"Estudo Genético de Factores de Predisposição para o Desenvolvimento e Progressão dos Cancros do Ovário e Endométrio"**, baseado na Metodologia observacional em casos e controlos, simultaneamente tranversal e longitudinal. Acresce que faço parte deste projecto e que este foi sujeito a concurso instituido pela Liga Portuguesa contra o Cancro em 1993, não tendo sido na altura financiado.

Com os melhores cumprimentos, ao dispor,

Clara Bicho

Dr. Maria Clara Bicho
