# Supplementary material for: Genetic Modulation of HPV Infection and Cervical Lesions: Role of Oxidative Stress-Related Genes
Source: Antioxidants (Basel). 2023 Sep 27;12(10):1806. doi: 10.3390/antiox12101806 (PMC10604255; doi:10.3390/antiox12101806)
Supplement: Supplementary file 1 [file antioxidants-12-01806-s001.zip › antioxidants-2525416-File S2 lei de 2005 - comissão de ética.pdf]

[Nº de artigos:**22**]

Lei n.º 12/2005, de 26 de Janeiro (versão actualizada)

INFORMAÇÃO GENÉTICA PESSOAL E INFORMAÇÃO DE SAÚDE

Contém as seguintes alterações:
- Lei n.º 26/2016, de 22 de Agosto

SUMÁRIO
Informação genética pessoal e informação de saúde

Lei n.º 12/2005, de 26 de Janeiro
Informação genética pessoal e informação de saúde
A Assembleia da República decreta, nos termos da alínea c) do artigo 161.º da Constituição, a lei seguinte:

Artigo 1.º
Objecto

A presente lei define o conceito de informação de saúde e de informação genética, a circulação de informação e a intervenção sobre o genoma humano no sistema de saúde, bem como as regras para a colheita e conservação de produtos biológicos para efeitos de testes genéticos ou de investigação.

Artigo 2.º
Informação de saúde

Para os efeitos desta lei, a informação de saúde abrange todo o tipo de informação directa ou indirectamente ligada à saúde, presente ou futura, de uma pessoa, quer se encontre com vida ou tenha falecido, e a sua história clínica e familiar.

Artigo 3.º
Propriedade da informação de saúde

1 - A informação de saúde, incluindo os dados clínicos registados, resultados de análises e outros exames subsidiários, intervenções e diagnósticos, é propriedade da pessoa, sendo as unidades do sistema de saúde os depositários da informação, a qual não pode ser utilizada para outros fins que não os da prestação de cuidados e a investigação em saúde e outros estabelecidos pela lei.
2 - O titular da informação de saúde tem o direito de, querendo, tomar conhecimento de todo o processo clínico que lhe diga respeito, salvo circunstâncias excepcionais devidamente justificadas e em que seja inequivocamente demonstrado que isso lhe possa ser prejudicial, ou de o fazer comunicar a quem seja por si indicado.
3 - O acesso à informação de saúde por parte do seu titular, ou de terceiros com o seu consentimento ou nos termos da lei, é exercido por intermédio de médico, com habilitação própria, se o titular da informação o solicitar.
4 - Na impossibilidade de apuramento da vontade do titular quanto ao acesso, o mesmo é sempre realizado com intermediação de médico.

Contém as alterações introduzidas pelos seguintes diplomas:
- Lei n.º 26/2016, de 22 de Agosto

Versões anteriores deste artigo:
- 1ª versão: Lei n.º 12/2005, de 26 de Janeiro

Artigo 4.º
Tratamento da informação de saúde

1 - Os responsáveis pelo tratamento da informação de saúde devem tomar as providências adequadas à protecção da sua confidencialidade, garantindo a segurança das instalações e equipamentos, o controlo no acesso à informação, bem como o reforço do dever de sigilo e da educação deontológica de todos os profissionais.
2 - As unidades do sistema de saúde devem impedir o acesso indevido de terceiros aos processos clínicos e aos sistemas informáticos que contenham informação de saúde, incluindo as respectivas cópias de segurança, assegurando os níveis de segurança apropriados e cumprindo as exigências estabelecidas pela legislação que regula a protecção de dados pessoais, nomeadamente para evitar a sua destruição, acidental ou ilícita, a alteração, difusão ou acesso não autorizado ou qualquer outra forma de tratamento ilícito da informação.
3 - A informação de saúde só pode ser utilizada pelo sistema de saúde nas condições expressas em autorização escrita do seu titular ou de quem o represente.
4 - O acesso a informação de saúde pode, desde que anonimizada, ser facultado para fins de investigação.
5 - A gestão dos sistemas que organizam a informação de saúde deve garantir a separação entre a informação de saúde e genética e a restante informação pessoal, designadamente através da definição de diversos níveis de acesso.

6 - A gestão dos sistemas de informação deve garantir o processamento regular e frequente de cópias de segurança da informação de saúde, salvaguardadas as garantias de confidencialidade estabelecidas por lei.

Artigo 5.º
Informação médica

1 - Para os efeitos desta lei, a informação médica é a informação de saúde destinada a ser utilizada em prestações de cuidados ou tratamentos de saúde.
2 - Entende-se por «processo clínico» qualquer registo, informatizado ou não, que contenha informação de saúde sobre doentes ou seus familiares.
3 - Cada processo clínico deve conter toda a informação médica disponível que diga respeito à pessoa, ressalvada a restrição imposta pelo artigo seguinte.
4 - A informação médica é inscrita no processo clínico pelo médico que tenha assistido a pessoa ou, sob a supervisão daquele, informatizada por outro profissional igualmente sujeito ao dever de sigilo, no âmbito das competências específicas de cada profissão e dentro do respeito pelas respectivas normas deontológicas.
5 - O processo clínico só pode ser consultado por médico incumbido da realização de prestações de saúde a favor da pessoa a que respeita ou, sob a supervisão daquele, por outro profissional de saúde obrigado a sigilo e na medida do estritamente necessário à realização das mesmas, sem prejuízo da investigação epidemiológica, clínica ou genética que possa ser feita sobre os mesmos, ressalvando-se o que fica definido no artigo 16.º

Artigo 6.º
Informação genética

1 - A informação genética é a informação de saúde que verse as características hereditárias de uma ou de várias pessoas, aparentadas entre si ou com características comuns daquele tipo, excluindo-se desta definição a informação derivada de testes de parentesco ou estudos de zigotia em gémeos, dos estudos de identificação genética para fins criminais, bem como do estudo das mutações genéticas somáticas no cancro.
2 - A informação genética pode ser resultado da realização de testes genéticos por meios de biologia molecular, mas também de testes citogenéticos, bioquímicos, fisiológicos ou imagiológicos, ou da simples recolha de informação familiar, registada sob a forma de uma árvore familiar ou outra, cada um dos quais pode, por si só, enunciar o estatuto genético de uma pessoa e seus familiares.
3 - A informação genética reveste natureza médica apenas quando se destina a ser utilizada nas prestações de cuidados ou tratamentos de saúde, no contexto da confirmação ou exclusão de um diagnóstico clínico, no contexto de diagnóstico pré-natal ou diagnóstico pré-implantatório ou no da farmacogenética, excluindo-se, pois, a informação de testes preditivos para predisposições a doenças comuns e pré-sintomáticos para doenças monogénicas.
4 - A informação genética que não tenha implicações imediatas para o estado de saúde actual, tal como a resultante de testes de paternidade, de estudos de zigotia em gémeos, e a de testes preditivos - com a excepção de testes genéticos para resposta a medicamentos -, de heterozigotia, pré-sintomáticos, pré-natais ou pré-implantatórios não pode ser incluída no processo clínico, salvo no caso de consultas ou serviços de genética médica com arquivos próprios e separados.
5 - Os processos clínicos de consultas ou serviços de genética médica não podem ser acedidos, facultados ou consultados por médicos, outros profissionais de saúde ou funcionários de outros serviços da mesma instituição ou outras instituições do sistema de saúde no caso de conterem informação genética sobre pessoas saudáveis.
6 - A informação genética deve ser objecto de medidas legislativas e administrativas de protecção reforçada em termos de acesso, segurança e confidencialidade.
7 - A utilização de informação genética é um acto entre o seu titular e o médico, que é sujeito às regras deontológicas de sigilo profissional dos médicos e dos restantes profissionais de saúde.
8 - A existência de vínculo laboral ou outro entre o médico ou outro profissional de saúde e qualquer actividade, incluindo companhias de seguros, entidades profissionais ou fornecedores de quaisquer bens ou serviços, não justifica qualquer diminuição aos deveres de segredo que sobre aqueles impendem.
9 - Os cidadãos têm o direito de saber se um processo clínico, ficheiro ou registo médico ou de investigação contém informação genética sobre eles próprios e a sua família e de conhecer as finalidades e usos dessa informação, a forma como é armazenada e os prazos da sua conservação.

Artigo 7.º
Bases de dados genéticos

1 - Entende-se por «base de dados genéticos» qualquer registo, informatizado ou não, que contenha informação genética sobre um conjunto de pessoas ou famílias.
2 - As regras de criação, manutenção, gestão e segurança das bases de dados genéticos para prestação de cuidados de saúde e relativas à investigação em saúde são regulamentadas nos termos da legislação que regula a protecção de dados pessoais.
3 - As bases de dados genéticos que contenham informação familiar e os registos genéticos que

permitam a identificação de familiares devem ser mantidas e supervisionadas por um médico com especialidade em genética ou, na sua falta, por outro médico.
4 - Qualquer pessoa pode pedir e ter acesso à informação sobre si própria contida em ficheiros com dados pessoais, nos termos da lei.

Artigo 8.º
Terapia genica

1 - A intervenção médica que tenha como objecto modificar intencionalmente o genoma humano só pode ser levada a cabo, verificadas as condições estabelecidas nesta lei, por razões preventivas ou terapêuticas.
2 - É proibida qualquer intervenção médica que tenha por objectivo a manipulação genética de características consideradas normais, bem como a alteração da linha germinativa de uma pessoa.

Artigo 9.º
Testes genéticos

1 - A realização de testes genéticos diagnósticos ou de farmacogenética obedece aos princípios que regem a prestação de qualquer cuidado de saúde.
2 - A detecção do estado de heterozigotia para doenças recessivas, o diagnóstico pré-sintomático de doenças monogénicas e os testes de susceptibilidades genéticas em pessoas saudáveis só podem ser executados com autorização do próprio, a pedido de um médico com a especialidade de genética e na sequência da realização de consulta de aconselhamento genético, após consentimento informado, expresso por escrito.
3 - A comunicação dos resultados de testes genéticos deve ser feita exclusivamente ao próprio, ou, no caso de testes diagnósticos, a quem legalmente o represente ou seja indicado pelo próprio, e em consulta médica apropriada.
4 - No caso de testes de estado de heterozigotia, pré-sintomáticos e preditivos, os resultados devem ser comunicados ao próprio e não podem nunca ser comunicados a terceiros sem a sua autorização expressa por escrito, incluindo a médicos ou outros profissionais de saúde de outros serviços ou instituições ou da mesma consulta ou serviço mas não envolvidos no processo de teste dessa pessoa ou da sua família.
5 - No caso de testes pré-natais e pré-implantatórios, os resultados devem ser comunicados exclusivamente à progenitora, aos progenitores ou aos respectivos representantes legais.
6 - Não devem ser realizados testes pré-sintomáticos, preditivos ou pré-implantatórios em pessoas com incapacidade mental que possam não compreender as implicações deste tipo de testes e dar o seu consentimento.
7 - Em situações de risco para doenças de início na vida adulta e sem cura nem tratamento comprovadamente eficaz, a realização do teste pré-sintomático ou preditivo tem ainda como condição uma avaliação psicológica e social prévia e o seu seguimento após a entrega dos resultados do teste.
8 - A frequência das consultas de aconselhamento genético e a forma do seguimento psicológico e social são determinadas considerando a gravidade da doença, a idade mais habitual de manifestação dos primeiros sintomas e a existência ou não de tratamento comprovado.

Artigo 10.º
Testes de heterozigotia, pré-sintomáticos, preditivos e pré-natais

1 - Para efeitos do artigo anterior, consideram-se testes para detecção do estado de heterozigotia os que permitam a detecção de pessoas saudáveis portadoras heterozigóticas para doenças recessivas.
2 - Consideram-se testes pré-sintomáticos os que permitam a identificação da pessoa como portadora, ainda assintomática, do genótipo inequivocamente responsável por uma dada doença monogénica.
3 - Consideram-se testes genéticos preditivos os que permitam a detecção de genes de susceptibilidade, entendida como uma predisposição genética para uma dada doença com hereditariedade complexa e com início habitualmente na vida adulta.
4 - Consideram-se testes de farmacogenética os testes preditivos que permitem a detecção de predisposições para respostas diferenciais no tratamento com um dado medicamento ou a susceptibilidade para reacções adversas derivadas da toxicidade da droga.
5 - Consideram-se testes pré-natais todos aqueles executados antes ou durante uma gravidez, com a finalidade de obtenção de informação genética sobre o embrião ou o feto, considerando-se assim como caso particular destes o diagnóstico pré-implantatório.
6 - Consideram-se testes de rastreio todos os testes diagnósticos, de heterozigotia, pré-sintomáticos, preditivos ou pré-natais que são aplicados a toda a população ou grupos populacionais de risco aumentado, nomeadamente por género, idade, origem étnica, em qualquer altura da vida.

Artigo 11.º
Princípio da não discriminação

1 - Ninguém pode ser prejudicado, sob qualquer forma, em função da presença de doença genética ou em função do seu património genético.
2 - Ninguém pode ser discriminado, sob qualquer forma, em função dos resultados de um teste genético diagnóstico, de heterozigotia, pré-sintomático ou preditivo, incluindo para efeitos de obtenção ou manutenção de emprego, obtenção de seguros de vida e de saúde, acesso ao ensino e, para efeitos de adopção, no que respeita quer aos adoptantes quer aos adoptandos.
3 - Ninguém pode ser discriminado, sob qualquer forma, nomeadamente no seu direito a seguimento médico e psicossocial e a aconselhamento genético, por se recusar a efectuar um teste genético.
4 - É garantido a todos o acesso equitativo ao aconselhamento genético e aos testes genéticos, salvaguardando-se devidamente as necessidades das populações mais fortemente atingidas por uma dada doença ou doenças genéticas.

Artigo 12.º

Testes genéticos e seguros

1 - As companhias de seguros não podem pedir nem utilizar qualquer tipo de informação genética para recusar um seguro de vida ou estabelecer prémios mais elevados.
2 - As companhias de seguros não podem pedir a realização de testes genéticos aos seus potenciais segurados para efeitos de seguros de vida ou de saúde ou para outros efeitos.
3 - As companhias de seguros não podem utilizar a informação genética obtida de testes genéticos previamente realizados nos seus clientes actuais ou potenciais para efeitos de seguros de vida e de saúde ou para outros efeitos.
4 - As seguradoras não podem exigir nem podem utilizar a informação genética resultante da colheita e registo dos antecedentes familiares para recusar um seguro ou estabelecer prémios aumentados ou para outros efeitos.

Artigo 13.º

Testes genéticos no emprego

1 - A contratação de novos trabalhadores não pode depender de selecção assente no pedido, realização ou resultados prévios de testes genéticos.
2 - Às empresas e outras entidades patronais não é permitido exigir aos seus trabalhadores, mesmo que com o seu consentimento, a realização de testes genéticos ou a divulgação de resultados previamente obtidos.
3 - Nos casos em que o ambiente de trabalho possa colocar riscos específicos para um trabalhador com uma dada doença ou susceptibilidade, ou afectar a sua capacidade de desempenhar com segurança uma dada tarefa, pode ser usada a informação genética relevante para benefício do trabalhador e nunca em seu prejuízo, desde que tenha em vista a protecção da saúde da pessoa, a sua segurança e a dos restantes trabalhadores, que o teste genético seja efectuado após consentimento informado e no seguimento do aconselhamento genético apropriado, que os resultados sejam entregues exclusivamente ao próprio e ainda desde que não seja nunca posta em causa a sua situação laboral.
4 - As situações particulares que impliquem riscos graves para a segurança ou a saúde pública podem constituir uma excepção ao anteriormente estipulado, observando-se no entanto a restrição imposta no número seguinte.
5 - Nas situações previstas nos números anteriores os testes genéticos, dirigidos apenas a riscos muito graves e se relevantes para a saúde actual do trabalhador, devem ser seleccionados, oferecidos e supervisionados por uma agência ou entidade independente e não pelo empregador.
6 - Os encargos da realização de testes genéticos a pedido ou por interesse directo de entidades patronais são por estas suportados.

Artigo 14.º

Testes genéticos e adopção

1 - Não podem ser pedidos testes genéticos, nem usada informação genética já disponível, para efeitos de adopção.
2 - Os serviços de adopção ou os pais prospectivos não podem pedir testes genéticos ou usar informação de testes anteriores nas crianças adoptandas.
3 - Os serviços de adopção não podem exigir aos pais adoptantes a realização de testes genéticos, nem usar informação já disponível sobre os mesmos.

Artigo 15.º

Laboratórios que procedem ou que oferecem testes genéticos

1 - Compete ao Governo regulamentar as condições da oferta e da realização de testes genéticos do estado de heterozigotia, pré-sintomáticos, preditivos ou pré-natais e pré-implantatórios, de modo a evitar, nomeadamente, a sua realização por laboratórios, nacionais ou estrangeiros, sem apoio de equipa médica e multidisciplinar necessária, assim como a eventual venda livre dos mesmos.
2 - Nos termos da lei e das recomendações éticas, de qualidade e de segurança dos organismos

reguladores nacionais e internacionais, o Governo determina medidas de acreditação e de certificação dos laboratórios públicos ou privados que realizem testes genéticos e procede ao seu licenciamento.

Artigo 16.º
Investigação sobre o genoma humano

1 - A investigação sobre o genoma humano segue as regras gerais da investigação científica no campo da saúde, estando obrigada a confidencialidade reforçada sobre a identidade e as características das pessoas individualmente estudadas.
2 - Deve ser garantido o livre acesso da comunidade científica aos dados emergentes da investigação sobre o genoma humano.
3 - A investigação sobre o genoma humano está sujeita à aprovação pelos comités de ética da instituição hospitalar, universitária ou de investigação.
4 - A investigação sobre o genoma humano em pessoas não pode ser realizada sem o consentimento informado dessas pessoas, expresso por escrito, após a explicação dos seus direitos, da natureza e finalidades da investigação, dos procedimentos utilizados e dos riscos potenciais envolvidos para si próprios e para terceiros.

Artigo 17.º
Dever de protecção

1 - É ilícita a criação de qualquer lista de doenças ou características genéticas que possa fundamentar pedidos de testes de diagnóstico, de heterozigotia, pré-sintomáticos, preditivos ou pré-natais ou de qualquer tipo de rastreio genético.
2 - Todo o cidadão tem direito a recusar-se a efectuar um teste genético do estado de heterozigotia, pré-sintomático, preditivo ou pré-natal.
3 - Todo o cidadão tem direito a receber aconselhamento genético e, se indicado, acompanhamento psicossocial, antes e depois da realização de testes de heterozigotia, pré-sintomáticos, preditivos e pré-natais.
4 - Só podem ser pedidos testes genéticos a menores desde que sejam efectuados em seu benefício e nunca em seu prejuízo, com o consentimento informado dos seus pais ou tutores, mas procurando-se sempre o seu próprio consentimento.
5 - Nomeadamente, não podem ser pedidos testes preditivos em menores para doenças de início habitual na vida adulta, sem prevenção ou cura comprovadamente eficaz.
6 - Do mesmo modo, o diagnóstico pré-natal para doenças de início habitual na vida adulta e sem cura não pode ser efectuado para mera informação dos pais, mas apenas para prevenção da doença ou deficiência, dentro dos prazos previstos na lei.
7 - Os médicos têm o dever de informar as pessoas que os consultam sobre os mecanismos de transmissão e os riscos que estes implicam para os seus familiares e de os orientar para uma consulta de genética médica, a qual deve ser assegurada nos termos da legislação regulamentar da presente lei.
8 - No caso dos testes de rastreio genético, deve sempre proteger-se, além dos direitos individuais, os direitos das populações ou grupos populacionais a rastrear, evitando-se a sua estigmatização.
9 - Os cidadãos com necessidades especiais, bem como os que são portadores de deficiências ou doenças crónicas, incluindo os doentes com patologias genéticas e seus familiares, gozam do direito à protecção do Estado em matéria de informação sobre os cuidados de saúde de que necessitam.

Artigo 18.º
Obtenção e conservação de material biológico

1 - A colheita de sangue e outros produtos biológicos e a obtenção de amostras de DNA para testes genéticos devem ser objecto de consentimento informado separado para efeitos de testes assistenciais e para fins de investigação em que conste a finalidade da colheita e o tempo de conservação das amostras e produtos deles derivados.
2 - O material armazenado é propriedade das pessoas em quem foi obtido e, depois da sua morte ou incapacidade, dos seus familiares.
3 - O consentimento pode ser retirado a qualquer altura pela pessoa a quem o material biológico pertence ou, depois da sua morte ou incapacidade, pelos seus familiares, devendo nesse caso as amostras biológicas e derivados armazenados ser definitivamente destruídos.
4 - Não devem ser utilizadas para efeitos assistenciais ou de investigação amostras biológicas cuja obtenção se destinou a uma finalidade diferente, a não ser com nova autorização por parte da pessoa a quem pertence ou, depois da sua morte ou incapacidade, dos seus familiares, ou após a sua anonimização irreversível.
5 - Amostras colhidas para um propósito médico ou científico específico só podem ser utilizadas com a autorização expressa das pessoas envolvidas ou seus representantes legais.
6 - Em circunstâncias especiais, em que a informação possa ter relevância para o tratamento ou a prevenção da recorrência de uma doença na família, essa informação pode ser processada e utilizada no contexto de aconselhamento genético, mesmo que já não seja possível obter o consentimento informado da pessoa a quem pertence.

7 - Todos os parentes em linha directa e do segundo grau da linha colateral podem ter acesso a uma amostra armazenada, desde que necessário para conhecer melhor o seu próprio estatuto genético, mas não para conhecer o estatuto da pessoa a quem a amostra pertence ou de outros familiares.
8 - É proibida a utilização comercial, o patenteamento ou qualquer ganho financeiro de amostras biológicas enquanto tais.

Artigo 19.º
Bancos de DNA e de outros produtos biológicos

1 - Para efeitos desta lei, entende-se por «banco de produtos biológicos» qualquer repositório de amostras biológicas ou seus derivados, com ou sem tempo delimitado de armazenamento, quer utilize colheita prospectiva ou material previamente colhido, quer tenha sido obtido como componente da prestação de cuidados de saúde de rotina, quer em programas de rastreio, quer para investigação, e que inclua amostras que sejam identificadas, identificáveis, anonimizadas ou anónimas.
2 - Ninguém pode colher ou usar amostras biológicas humanas já colhidas ou seus derivados, com vista à constituição de um banco de produtos biológicos, se não tiver obtido autorização prévia de entidade credenciada pelo departamento responsável pela tutela da saúde, assim como da Comissão Nacional de Protecção de Dados se o banco estiver associado a informação pessoal.
3 - Os bancos de produtos biológicos devem ser constituídos apenas com a finalidade da prestação de cuidados de saúde, incluindo o diagnóstico e a prevenção de doenças, ou de investigação básica ou aplicada à saúde.
4 - Um banco de produtos biológicos só deve aceitar amostras em resposta a pedidos de médicos e não das próprias pessoas ou seus familiares.
5 - O consentimento informado escrito é necessário para a obtenção e utilização de material para um banco de produtos biológicos, devendo o termo de consentimento incluir informação sobre as finalidades do banco, o seu responsável, os tipos de investigação a desenvolver, os seus riscos e benefícios potenciais, as condições e a duração do armazenamento, as medidas tomadas para garantir a privacidade e a confidencialidade das pessoas participantes e a previsão quanto à possibilidade de comunicação ou não de resultados obtidos com esse material.
6 - No caso de uso retrospectivo de amostras ou em situações especiais em que o consentimento das pessoas envolvidas não possa ser obtido devido à quantidade de dados ou de sujeitos, à sua idade ou outra razão comparável, o material e os dados podem ser processados, mas apenas para fins de investigação científica ou obtenção de dados epidemiológicos ou estatísticos.
7 - A conservação de amostras de sangue seco em papel obtidas em rastreios neonatais ou outros deve ser considerada à luz dos potenciais benefícios e perigos para os indivíduos e a sociedade, podendo, no entanto, essas colecções ser utilizadas para estudos familiares no contexto do aconselhamento genético ou então para investigação genética, desde que previamente anonimizadas de forma irreversível.
8 - Deve ser sempre garantida a privacidade e a confidencialidade, evitando-se o armazenamento de material identificado, controlando-se o acesso às colecções de material biológico, limitando-se o número de pessoas autorizadas a fazê-lo e garantindo-se a sua segurança quanto a perdas, alteração ou destruição.
9 - Só podem ser usadas amostras anónimas ou irreversivelmente anonimizadas, devendo as amostras identificadas ou identificáveis ficar limitadas a estudos que não possam ser feitos de outro modo.
10 - Não é permitido o armazenamento de material biológico humano não anonimizado por parte de entidades com fins comerciais.
11 - Havendo absoluta necessidade de se usarem amostras identificadas ou identificáveis, estas devem ser codificadas, ficando os códigos armazenados separadamente, mas sempre em instituições públicas.
12 - Se o banco envolver amostras identificadas ou identificáveis e estiver prevista a possibilidade de comunicação de resultados dos estudos efectuados, deve ser envolvido nesse processo um médico especialista em genética.
13 - O material biológico armazenado é considerado propriedade da pessoa de quem foi obtido ou, depois da sua morte ou incapacidade, dos seus familiares, devendo ser armazenado enquanto for de comprovada utilidade para os familiares actuais e futuros.
14 - Os investigadores responsáveis por estudos em amostras armazenadas em bancos de produtos biológicos devem sempre verificar que os direitos e os interesses das pessoas a quem o material biológico pertence são devidamente protegidos, incluindo a sua privacidade e confidencialidade, mas também no que respeita à preservação das amostras, que podem mais tarde vir a ser necessárias para diagnóstico de doença familiar, no contexto de testes genéticos nessas pessoas ou seus familiares.
15 - Compete aos investigadores responsáveis pela colecção e manutenção de bancos de produtos biológicos zelar pela sua conservação e integridade e informar as pessoas de quem foi obtido consentimento de qualquer perda, alteração ou destruição, assim como da sua decisão de abandonar um tipo de investigação ou de fechar o banco.
16 - A lei define as regras para o licenciamento e a promoção de processos de garantia de qualidade dos bancos de produtos biológicos.
17 - A transferência de um grande número de amostras ou colecções de material biológico para outras entidades nacionais ou estrangeiras deve sempre respeitar o propósito da criação do banco para o qual foi obtido o consentimento e ser aprovada pelas comissões de ética responsáveis.
18 - A constituição de bancos de dados que descrevam uma determinada população e a eventual transferência dos seus dados devem ser aprovadas pelo Conselho Nacional de Ética para as Ciências

da Vida e, no caso de serem representativos da população nacional, pela Assembleia da República.
19 - Os bancos de produtos biológicos constituídos para fins forenses de identificação criminal ou outros devem ser objecto de regulamentação específica.

Artigo 20.º
Património genético humano

O património genético humano não é susceptível de qualquer pantenteamento.

Artigo 21.º
Relatório sobre a aplicação da lei

O Governo, ouvido o Conselho Nacional de Ética para as Ciências da Vida, apresenta à Assembleia da República, no prazo de dois anos após a entrada em vigor desta lei, e a cada dois anos subsequentes, um relatório que inventarie as condições e as consequências da sua aplicação, considerando a evolução da discussão pública acerca dos seus fundamentos éticos e os progressos científicos entretanto obtidos.

Artigo 22.º
Regulamentação

1 - Compete ao Governo a regulamentação desta lei no prazo de 180 dias.
2 - É objecto de regulamentação própria a definição de medidas de promoção da investigação e de protecção da identidade genética pessoal, de validação clínica e analítica dos testes genéticos, particularmente dos testes preditivos para genes de susceptibilidade e da resposta a tratamentos medicamentosos, bem como dos testes de rastreio genético.

Aprovada em 9 de Dezembro de 2004.
O Presidente da Assembleia da República, João Bosco Mota Amaral.
Promulgada em 7 de Janeiro de 2005.
Publique-se.
O Presidente da República, JORGE SAMPAIO.
Referendada em 13 de Janeiro de 2005.
O Primeiro-Ministro, Pedro Miguel de Santana Lopes.
