# Supplementary material for: Genetic Modulation of HPV Infection and Cervical Lesions: Role of Oxidative Stress-Related Genes
Source: Antioxidants (Basel). 2023 Sep 27;12(10):1806. doi: 10.3390/antiox12101806 (PMC10604255; doi:10.3390/antiox12101806)
Supplement: Supplementary file 1 [file antioxidants-12-01806-s001.zip › antioxidants-2525416-Table S1.pdf]

Supplementary Table S1. Comparison of genotypes distribution – HSIL&ICC *vs* Control.

**Gene**		**HSIL&ICC N (%)**	**CONTROLS N (%)**	**p-value[a]**	**OR [CI]**
ACE1	ID&II	23 (35.4%)	209 (54.3%)	**0.005**	0.461 [0.267-0.797]
	DD	42 (64.6%)	176 (45.7%)		

[a] Chi-square test
